# Trends in maternal body mass index, macrosomia and caesarean section in first-time mothers during the pandemic: a multicentre retrospective cohort study of 12 Melbourne public hospitals

**DOI:** 10.1186/s12884-024-06908-y

**Published:** 2024-10-28

**Authors:** Andrew J. Goldsack, Melvin B. Marzan, Daniel L. Rolnik, Anthea C. Lindquist, Joanne M. Said, Kirsten R. Palmer, Penelope M. Sheehan, Stephanie Potenza, Natasha Pritchard, Clare L. Whitehead, Jolyon Ford, Ben W. Mol, Susan P. Walker, Lisa Hui

**Affiliations:** 1 Melbourne Medical School, The University of Melbourne, Melbourne, Australia; 2 Department of Obstetrics, Gynaecology and Newborn Health, Melbourne Medical School, The University of Melbourne, Melbourne, Australia; 3 Department of Obstetrics and Gynaecology, Mercy Hospital for Women, Mercy Health, Melbourne, Australia; 4 Reproductive Epidemiology Group, Murdoch Children’s Research Institute, Melbourne, Australia; 5 Department of Obstetrics and Gynaecology, Monash Health, Melbourne, Australia; 6 Department of Obstetrics and Gynaecology, Monash University, Melbourne, Australia; 7 Maternal-Fetal Medicine Department, Joan Kirner Women’s and Children’s Hospital, Western Health, Melbourne, Australia; 8 Department of Obstetrics and Gynaecology, The Royal Women’s Hospital, Melbourne, Australia; 9 Department of Obstetrics and Gynaecology, Eastern Health, Melbourne, Australia; 10 Department of Obstetrics and Gynaecology, Peninsula Health, Melbourne, Australia; 11 Department of Obstetrics and Gynaecology, The Northern Hospital, Northern Health, Melbourne, Australia

**Keywords:** Obesity, COVID-19, Pregnancy, Birthweight, Pregnancy complications

## Abstract

**Objective:**

To compare specific perinatal outcomes in nulliparas with a singleton infant in cephalic presentation at term, with and without exposure to the COVID-19 pandemic during pregnancy. We hypothesised that the pandemic conditions in Melbourne may have been an independent contributor to trends in maternal Body Mass Index ≥ 25 kg/m^2^, macrosomia and caesarean section.

**Design:**

Multi-centre retrospective cohort study and interrupted time-series analysis.

**Setting:**

Metropolitan Melbourne, Victoria.

**Population:**

Singleton infants ≥ 20 weeks gestational age born between 1 January 2019 and 31 March 2022.

**Main outcome measures:**

Rates of maternal Body Mass Index ≥ 25 kg/m^2^, macrosomia (birthweight *≥* 4000 g) and caesarean section.

**Results:**

25 897 individuals gave birth for the first time to a singleton infant in cephalic presentation at term in the pre-pandemic cohort, and 25 298 in the pandemic-exposed cohort. Interrupted time-series analysis demonstrated no significant additional effect of the pandemic on pre-existing upward trends in maternal Body Mass Index ≥ 25 kg/m^2^, caesarean section or macrosomia. The rate of maternal Body Mass Index ≥ 25 kg/m^2^ was higher in the pandemic-exposed cohort compared with the pre-pandemic cohort, (45.82% vs. 44.58% respectively, *p* = 0.041) as was the overall rate of caesarean section (33.09% vs. 30.80%, *p* < 0.001). However, this increase in caesarean section was confined to individuals who had either an induction of labour or no labour. There was also a nonsignificant trend to higher rates of macrosomia in the pandemic-exposed cohort compared with the pre-pandemic cohort (8.55% vs. 7.99% respectively, *p* = 0.124).

**Conclusions:**

While rates of Body Mass Index ≥ 25 kg/m^2^, pre-labour caesarean section, and caesarean section following induction of labour were higher among pandemic-exposed nulliparas, these findings represented a continuation of pre-existing upward trends, with no significant independent contribution from the pandemic. These trends are forecast to continue, with long term implications for population health.

**Supplementary Information:**

The online version contains supplementary material available at 10.1186/s12884-024-06908-y.

## Introduction

The obesity and overweight epidemic in Australia is a major public health priority for our healthcare system [1]. Australian studies have reported that the COVID-19 pandemic may have influenced the weight of some populations, with overweight and obesity (BMI ≥ 25 kg/m^2^) being more common among all age groups except the elderly [2, 3]. A multicentre study of all births in Melbourne public hospitals showed an increase in the proportion of pregnant individuals with a BMI ≥ 25 kg/m^2^ during the pandemic [4]. Higher maternal weight during pregnancy is associated with higher rates of delivery by caesarean section (CS) and confers increased risks of obstetric complications such as gestational diabetes, and macrosomic birthweight ≥ 4000 g. Maternal weight also has implications for the next generation through epigenetic modification of the infant [5], and has been associated with childhood obesity and adverse metabolic profiles [6–8]. Over the past 25 years, a greater proportion of people giving birth have had a BMI *≥* 25 kg/m^2^. Prevention of the associated adverse maternal and childhood outcomes through strategies such as optimisation of pre-pregnancy maternal weight is an important public health priority [9]. 

CS has important implications for subsequent pregnancies, including the risk of placental adhesive disorder and uterine rupture, health systems and resources as well as impacting newborn health [6, 10]. Safely mitigating the rise in CS has therefore become a global focus in obstetric care [11]. Individuals giving birth for the first time (nulliparas) are considered a high priority group for addressing the rising CS rate, as CS in a first pregnancy makes subsequent deliveries by CS more likely [12]. 

Authors [13] have described the relationship between the COVID-19 pandemic and obesity by noting the psychological impact, difficulty accessing healthcare and limitations to physical activity as contributors to the global obesity crisis. Metropolitan Melbourne had 18 months of government mandates restricting movement of people during the COVID-19 pandemic, accompanied by abrupt changes in the provision of routine antenatal care. These factors may have influenced maternal weight and associated obstetric complications during 2020–2021 [13]. 

The objective of this study was to analyse trends of maternal BMI *≥* 25 kg/m^2^, macrosomia and CS before and during the pandemic. We hypothesised that the pandemic and the associated lockdown restrictions made an independent contribution to the rates of maternal BMI *≥* 25 kg/m^2^, macrosomia and CS.

## Methods

### Study design

We conducted a multi-centre retrospective cohort analysis of perinatal data in two parts: (i) an interrupted time-series analysis (ITSA) of perinatal outcomes, with a forecast based on pre-exposure trends, and (ii), summary statistics (n and %) of maternal BMI *≥* 25 kg/m^2^, macrosomia, and CS births in groups and Poisson regression analysis comparing cohorts with and without exposure to the pandemic. Macrosomia is defined as birthweight ≥ 4000 g, rather than by centile for gestational age (GA), as our population of interest is first-time mothers delivering at or after 37 weeks GA.

### Study population and data sources

Non-identifiable data was obtained from routine birth data collection systems with a waiver of consent. This analysis includes all births of ≥ 20 weeks GA from all 12 public maternity hospitals in Melbourne from 1 January 2018 to 31 March 2022. Data from private maternity hospitals in Melbourne were not available for this study. The twelve hospitals capture approximately 78% of all births from Melbourne and include all four tertiary maternity units [14]. 

### Inclusion and exclusion criteria

Singleton births of infants ≥ 20 weeks GA and classifiable in the Robson classification system were included [15, 16]. Exclusions were: congenital abnormalities, deliveries < 20 weeks GA or with unknown GA, terminations of pregnancy, non-Victorian residents and births with missing or contradictory information in the variables needed for Robson classification [17]. 

### Definition of Robson 1, 2A and 2B

The Robson classification system is a global standard for describing birth cohorts to facilitate standardised comparison of CS rates within and between healthcare systems [18]. In this study, we focussed on Robson groups 1, 2A and 2B as these represent individuals for whom averting a CS has the highest potential benefit for individual health outcomes and healthcare systems.

- **Robson 1**: Nulliparous, singleton, cephalic presentation, ≥ 37 week GA pregnancies where labour commenced spontaneously. This group includes individuals who received oxytocin or had an amniotomy for augmentation of labour.
- **Robson 2A**: Nulliparous, singleton, cephalic presentation, ≥ 37 week GA pregnancies for whom labour was induced.
- **Robson 2B**: Nulliparous, singleton, cephalic presentation, ≥ 37 week GA pregnancies that delivered without labour, (i.e. pre-labour CS) [15, 16].

### Outcomes

Our primary outcomes are reported as frequency and rates;

1. Proportion of mothers with BMI *≥* 25 kg/m^2^.
2. Proportion of infants delivered with birthweight ≥ 4000 g (macrosomia).
3. Proportion of infants delivered by caesarean section. 
4. Proportion of infants with birthweight ≥ 4000 g (macrosomia), delivered by caesarean section.

### Pandemic exposure definitions

**Fig. 1:**
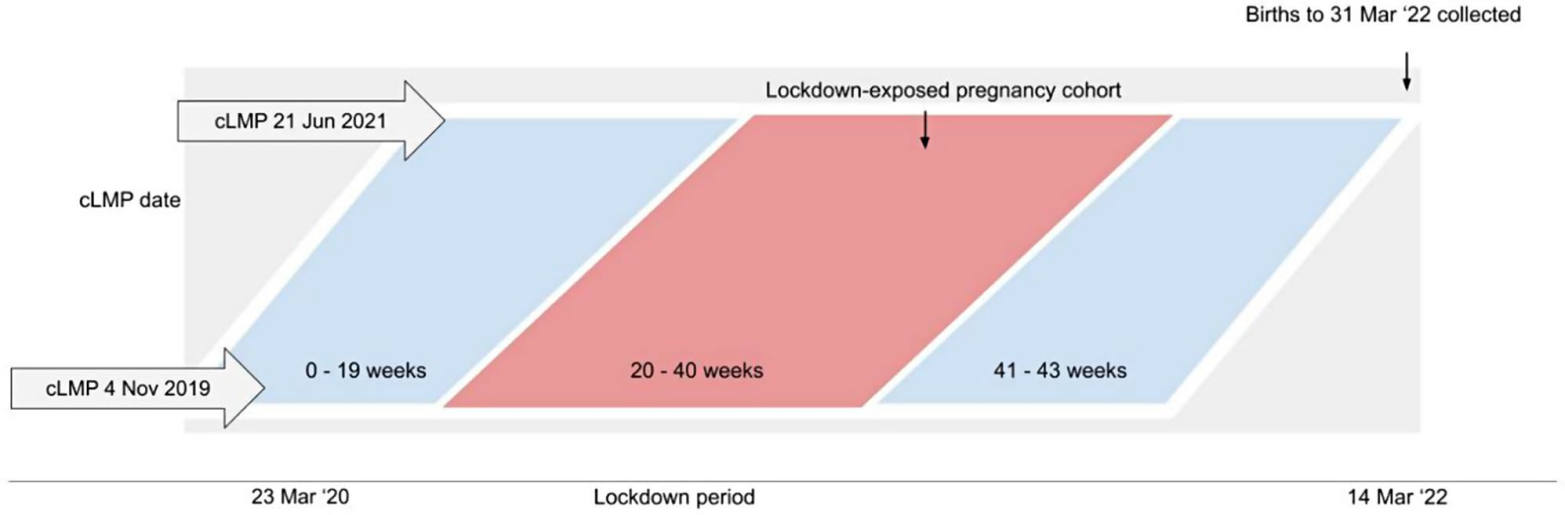
Pandemic-exposed group timeline. cLMP = calculated week of last menstrual period. *The red bar indicates the cohort exposed to pandemic/pandemic lockdowns during weeks 20–40 of gestation. The cLMP of week of 4 November 2019 was made as the reference point to define the pregnancies were exposed to pandemic conditions commencing 23 March 2020 from no later than 20 weeks gestation

Gestational exposure to pandemic conditions is a time-dependent exposure. To avoid the fixed cohort bias [19] that arises from using calendar dates to define study cohorts with time-dependent exposures, we used ‘calculated week of last menstrual period’ (cLMP) rather than week-of-birth, as previously described [20], to define the pandemic-exposed cohort and ensure all pregnancies in the pandemic cohort had an equivalent duration of exposure (Fig. 1).

For privacy protection, hospital data managers converted the actual infant date-of-birth into the ordinal calendar week-of-birth (i.e. 1 to 52 for each calendar week). To generate the cLMP, we used the week-of-birth and gestational age in completed weeks at delivery. The formula used was:

$$\tt \eqalign{& First{\rm{ }}\:day{\rm{ }}\:of{\rm{ }}\:the{\rm{ }}\:week\; of\; cLMP \cr & = week\; of\; birth - \left[ {GA\left( {in{\rm{ }}\:completed{\rm{ }}\:weeks} \right) \times 7} \right] \cr}$$

Using this cLMP, we defined a ‘pandemic-exposed’ cohort comprising women for whom weeks 20–40 of gestation would have occurred during the lockdown period. (Fig. 1) With a defined pandemic-exposure from 23 March 2020 (Monday of the week of suspension of elective surgery due to COVID-19 in Victoria) to 28 March 2022 (which formed a 2 year period of pandemic-exposure), this included women whose cLMP occurred during the 85 weeks from 4 November 2019 to 21 June 2021 inclusive [16]. Pre-labour caesarean sections for medical indications were not suspended in Victoria during this time. The pre-pandemic group comprised women who had their cLMP during the corresponding calendar weeks commencing two years prior to the start of the exposed cohort (births with cLMPs in weeks commencing 30 October 2017 to 3 June 2019). We have included gestational ages from 20 to 40 weeks in both the pandemic-exposed and pre-pandemic cohorts. This approach ensures that the cohorts were adequately exposed to the effects of either the pre-pandemic or pandemic conditions, facilitating meaningful comparison of the outcomes.

### Statistical analysis

We conducted two statistical analyses on the population of interest. The main analysis is the interrupted time-series analysis and the pre-specified complimentary cohort analysis is considered secondary and hypothesis generating.

(i) Interrupted time-series analysis (ITSA) of weekly rates of maternal BMI *≥* 25 kg/m^2^, macrosomia and CS births in Robson groups 1 and 2.

We conducted ITSA using the cLMP from 30 October 2017 to 28 March 2022. Our intervention period started from cLMP of 4 November 2019. We employed ITSA using the “itsa” suite of commands in Stata 18 [21]. This approach used the Prais-Winstein generalised least-squares regression, accounting for autocorrelation of the residuals [21]. We used sine and cosine functions to correct for seasonality [21]. (Supplemental File 1)

(ii) Cohort analysis of the proportions of maternal BMI ≥ 25 kg/m^2^, macrosomia and CS births in Robson groups 1 and 2 before and during the pandemic.

We present the primary outcomes as frequency (n) and percentage/proportion (%) in accordance with the World Health Organisation’s Robson Manual recommendations [15, 16]. Statistical significance was assessed using independent samples t-tests or Chi-square tests as appropriate. The analyses were conducted in Stata version 18 [22], with two-tailed p-values below 0.05 considered statistically significant. To compare the pandemic cohort with the pre-pandemic cohort, we independently employed Poisson regression, adjusting for covariates such as maternal age, maternal BMI, maternal region of birth, smoking status, socioeconomic status, and requirement for an interpreter. Covariates were selected based on a priori and subject matter expertise. The effect estimates were reported as adjusted relative risk (aRR) with 95% confidence intervals (CI). We accounted for the observations with missing data on covariates by using the multiple imputation by chained equation (MICE) using the “mi impute chained” command in Stata 18 [23]. 

### Ethical approval

Ethical approval was obtained from Austin Health (HREC/64722/Austin-2020) and Mercy Health Ethics Committees (ref. 2020-031).

## Results

### Main analysis: interrupted time-series analysis (ITSA) of Robson groups 1 & 2

**Fig. 2:**
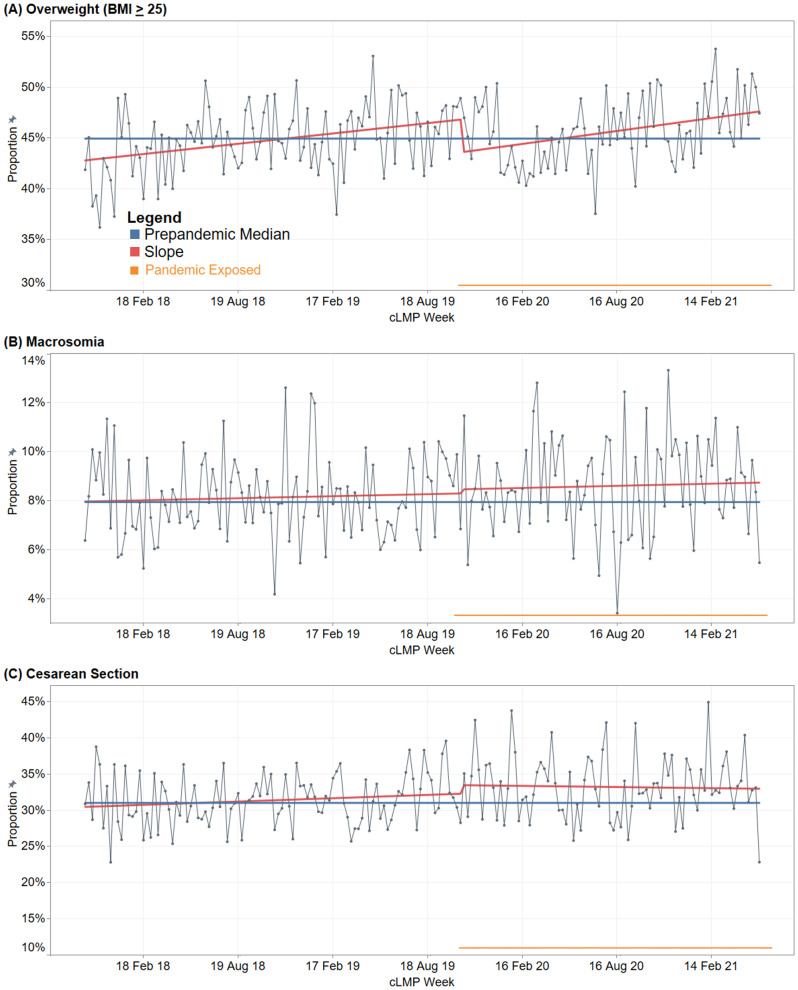
Maternal BMI ≥ 25 **(A)**, fetal macrosomia **(B)** and caesarean sections **(C)** for Robson groups 1 & 2 from October 30 2017 to June 21 2021 time series analysis by cLMP. cLMP = calculated last menstrual period. * Adjusted for autocorrelation and seasonality. Median pre-pandemic values: (A) Overweight 42.79% (interquartile range [IQR], 42.61–46.99%), (B) Macrosomia, 7.97% (IQR, 6.89–9.25%) and (C) Caesarean Sect. 30.47% (IQR, 28.98–33.89%). ** Orange line indicates the cohort exposed to pandemic/pandemic lockdowns with a defined pandemic-exposure, cLMP of week of 4 November 2019 was made as the reference point which means babies were adequately exposed to pandemic lockdown effects that started 23 March 2020

The ITSA results are shown in Fig. 2; Table 1. Importantly, there was a pre-existing upward trend in the rate of maternal BMI ≥ 25 kg/m^2^ prior to the onset of the pandemic at 0.04% per week (95% CI: 0.01–0.06%). Pandemic exposure was not associated with a significant change in the rate of rise in pregnant individuals with BMI *≥* 25 kg/m^2^ (Fig. 2; Table 1). Similarly, the pre-existing uptrends in macrosomia and CS did not change significantly following the onset of the pandemic (Fig. 2; Table 1).

### Secondary analysis: cohort analysis of Robson groups 1 & 2

**Table 1:** Trends in BMI *≥* 25, macrosomia and CS among Robson groups 1 & 2 in the pre-pandemic and pandemic-exposed cohorts

Variable	Slope in per cent (95% confidence interval)	
	Pre-pandemic period (30 Oct 2017–28 Oct 2019 )	Pandemic-exposed period (4 Nov 2019–21 Jun 2021)
Overweight (BMI *≥* 25)	0.04 (0.01 to 0.06)	0.01 (–0.02 to 0.04)
Macrosomia	0.004 (–0.01 to 0.01)	0.001 (–0.02 to 0.02)
Caesarean section	0.02 (-0.01 to 0.04)	–0.03 (–0.07 to 0.02)

### Nulliparas with a term, singleton cephalic fetus (Robson groups 1 & 2)

**Fig. 3:**
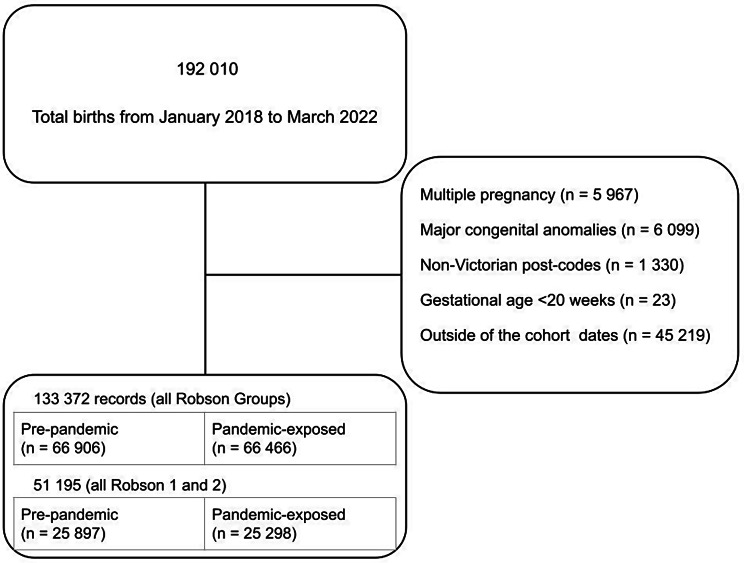
Analysis flow-chart

Pre-pandemic and pandemic-exposed nulliparas with term, cephalic, singleton fetuses (Robson groups 1 and 2) were compared. There were 25 897 (50.6%) in the pre-pandemic cohort and 25 298 (49.4%) in the pandemic-exposed cohort (Fig. 3); baseline characteristics are provided in Table 2. The maternal and neonatal characteristics of Robson groups 1 and 2 were similar to those of the overall study population.

**Table 2:** Characteristics of pre-pandemic and pandemic cohorts (Robson groups 1 & 2)

Characteristic	Pre-pandemic period (cLMP 20 Oct ’17–3 Jun ’19)	Pandemic-exposed period (cLMP 4 Nov ’19–21 Jun ’21)
Total Births (%)	25 897 (50.6)	25 298 (49.4)
Maternal Weight in Kg, mean (SD)***	68.6 (16.5)	69.2 (16.4)
Maternal Height in cm, mean (SD)	163.3 (7.0)	163.7 (7.2)
Birth Weight in grams, mean (SD)**	3352.3 (461.9)	3365.8 (490.0)
Gestational Age at weeks, mean (SD)**	39.49 (1.2)	39.53 (1.2)
Maternal age group (years), n (%)***		
18–24	4 767 (18.4)	3 995 (15.8)
25–29	7 321 (28.3)	6 746 (26.7)
30–34	9 647 (37.3)	10 122 (40.0)
35–39	3 479 (13.4)	3 726 (14.7)
40 or older	683 (2.6)	652 (2.6)
Missing	0 (0.0)	57 (0.2)
BMI Categories, n (%)*		
<18	429 (1.7)	390 (1.5)
18–24	13 807 (53.3)	12 818 (50.7)
25–29	7 035 (27.2)	6 748 (26.7)
30–34	2 625 (10.1)	2 676 (10.6)
35–39	1 099 (4.2)	1 080 (4.3)
≥40	686 (2.6)	665 (2.6)
Missing	216 (0.8)	921 (3.6)
Socio-economic status (IRSAD quintile), n (%) *		
1 (most disadvantaged)	5 147 (20.0)	4 925 (19.5)
2	3 847 (15.0)	3 705 (14.7)
3	5 668 (21.9)	5 788 (22.9)
4	5 962 (23.0)	5 937 (23.5)
5 (most advantaged)	5 219 (20.1)	4 943 (19.4)
Region of birth, n (%)***		
Australia and associated territories	13 429 (51.9)	14 068 (55.6)
Americas	441 (1.7)	519 (2.1)
North Africa and the Middle East	886 (3.4)	700 (2.8)
North-East Asia	1 294 (5.0)	933 (3.7)
North-West Europe	894 (3.5)	933 (3.7)
Oceania including New Zealand	816 (3.2)	778 (3.1)
South-East Asia	2 181 (8.4)	1 911 (7.6)
Southern and Central Asia	4 756 (18.4)	4 316 (17.1)
Southern and Eastern Europe	558 (2.2)	483 (1.9)
Sub-Saharan Africa	545 (2.1)	480 (1.9)
Missing	97 (0.4)	177 (0.7)
Smoking, n (%) **		
Yes	1 121 (4.3)	955 (3.8)
Gestational age at birth, n (%)***		
37–41	25 763 (99.5)	25 055 (99.0)
42+	134 (0.5)	243 (1.0)

IRSAD = Index of Relative Socio-economic Advantage and Disadvantage

Statistical Significance * < 0.05, **<0.01, ***<0.001

N.B. Several categorical variable (%) may not sum to 100% due to rounding

Table 3 shows outcomes of interest by Robson group. The rate of maternal BMI ≥ 25 kg/m^2^ was significantly higher among term nulliparas with a cephalic singleton fetus in the pandemic-exposed cohort compared with the pre-pandemic cohort (45.82% vs. 44.58%, *p* = 0.041). The rate of CS was also higher in the pandemic-exposed cohort compared with the pre-pandemic cohort (33.09% vs. 30.80%, *p* < 0.005). However, the rate of macrosomia did not significantly differ between the pandemic-exposed and pre-pandemic cohorts (8.55% and 7.99%, *p* = 0.124).

**Table 3:** Outcomes by Robson groups 1 & 2

Outcomes by Robson groups *N* (%)	Group		Adjusted relative risk (aRR)† (95% Confidence Interval)
	Pre-pandemic period (cLMP 20 Oct ’17–3 Jun ’19)	Pandemic-exposed period (cLMP 4 Nov ’19–21 Jun ’21)	
**Robson 1 & 2**			
Robson 1 & 2 births, n (% all Robson groups)	25 897 (38.71)	25 298 (38.06)	0.98 (0.96 to 0.999)*
Macrosomic infants, n (% Robson 1 & 2)	2 070 (7.99)	2 162 (8.55)	1.05 (0.99 to 1.12)
CS births, n (% Robson 1 & 2)	7 977 (30.80)	8 372 (33.09)	1.07 (1.03 to 1.10)***
CS rate among macrosomic infants, n (% Robson 1 & 2)	923 (44.59)	1 062 (49.12)	1.16 (1.06 to 1.27)**
Maternal BMI ≥ 25 (%)	11 446 (44.58)	11 169 (45.82)	1.02 (1.00 to 1.03)*
**Robson 1**			
Robson 1 births, n (% all Robson groups)	10 813 (16.16)	10 496 (16.47)	1.06 (1.04 to 1.09)***
Robson 1 births, n (% of Robson 1 & 2 births)	10 813 (41.75)	10 496 (41.49)	1.03 (1.01 to 1.06)*
Macrosomic infants, n (% Robson 1)	794 (7.34)	838 (7.66)	1.02 (0.92 to 1.13)
CS births, n (% Robson 1)	1 815 (16.79)	1 855 (16.95)	1.00 (0.94 to 1.07)
CS rate among macrosomic infants, n (% Robson 1)	210 (26.45)	252 (30.07)	1.18 (0.98 to 1.43)
**Robson 2A**			
Robson 2A births, n (% all Robson groups)	13 843 (20.69)	12 763 (19.20)	0.96 (0.94 to 0.99)**
Robson 2A births, n (% of Robson 1 & 2 births)	13 843 (53.45)	12 763 (50.45)	0.95 (0.92 to 0.97)***
Macrosomic infants, n (% Robson 2A)	1 147 (8.29)	1 161 (9.10)	1.08 (0.99 to 1.17)
CS births, n (% Robson 2A)	4 921 (35.55)	4 928 (38.61)	1.08 (1.04 to 1.12)***
CS rate among macrosomic infants, n (% Robson 2A)	584 (50.92)	647 (55.73)	1.18 (1.04 to 1.32)***
**Robson 2B**			
Robson 2B births, n (% all Robson groups)	1 241 (1.85)	1 589 (2.39)	1.27 (1.18 to 1.38)***
Robson 2B births, n (% of Robson 1 & 2 births)	1 241 (4.79)	1 589 (6.28)	1.26 (1.17 to 1.37)***
Macrosomic infants, n (% Robson 2B)	129 (10.39)	163 (10.26)	0.97 (0.76 to 1.24)
CS births, n (% Robson 2B)	1 241 (100.00)	1 589 (100.00)	-
CS rate among macrosomic infants, n (% Robson 2B)	129 (100.00)	163 (100.00)	-

Missing data was accounted by multiple imputation by chained equation (MICE)

Statistical Significance * < 0.05, **<0.01, ***<0.001

† - adjusted for maternal country of birth, maternal smoking, socioeconomic status, baby sex, pertussis vaccination, and interpreter requirement

Robson 1 & 2: Nulliparas with singleton, term, cephalic births

Robson 1: Nulliparas with singleton, term, cephalic births after spontaneous onset of labour

Robson 2A: Nulliparas with singleton, term, cephalic births following induction of labour

Robson 2B: Nulliparas with singleton, term, cephalic births without labour

There was no significant change in the proportion of births by CS following spontaneous onset of labour in the pandemic-exposed cohort (Robson 1). There was a greater proportion of births by CS following induction of labour (Robson 2A) in the pandemic-exposed cohort compared with the pre-pandemic cohort (38.61% vs. 35.55%, *p* < 0.005). There was also a greater proportion of pre-labour CS for nulliparas with term, singleton, cephalic fetuses (Robson 2B) in the pandemic-exposed cohort compared with the pre-pandemic cohort (2.39% vs. 1.85%, *p* < 0.005).

## Discussion

Our study is one of few examining the relationship between the pandemic, BMI and obstetric outcomes. We demonstrated using ITSA that the higher rates of maternal BMI ≥ 25 kg/m^2^ and CS observed in first-time mothers exposed to the pandemic were continuations of pre-existing trends that were not accelerated during the pandemic.

Anderson et al. [24] reported that the global prevalence of obesity in the general adult population increased by 1% during the pandemic. However, a systematic review and meta-analysis [25] of the impact of the COVID-19 pandemic on perinatal outcomes made no comment on maternal BMI or macrosomia. There are conflicting reports on the rate of CS during the pandemic, with some concluding that there was no difference in high-income countries [25], while others observed a significant reduction in primary CS [26]. Our study, using both ITSA and an exploratory cohort analysis, provides confirmation of higher rates of these outcomes during the pandemic, while clarifying the lack of independent contribution from the pandemic.

At face value, the proportion of maternal BMI ≥ 25 kg/m² in first-time mothers appears to decrease at the onset of the pandemic, but it has continued to increase since then, albeit at a slower rate compared to the pre-pandemic period (Fig. 2). We can only speculate as to the reason for this initial trend and it is likely influenced by seasonality patterns and statistical variation rather than a direct or indirect association with the COVID-19 pandemic, as indicated by the non-significant slope coefficient. This longstanding uptrend in maternal BMI *≥* 25 kg/m^2^ portends higher rates of obstetric, neonatal and childhood consequences. Maternal weight is a modifiable risk factor, so promotion of a healthy diet and regular exercise, ideally before conception, is an important approach to address these outcomes. A trial of preconception weight optimisation is currently underway in New South Wales, which may provide evidence for future interventions to reduce the perinatal and childhood morbidity associated with higher maternal BMI [27]. 

Despite a higher proportion of maternal BMI *≥* 25 kg/m^2^ during the pandemic, fetal macrosomia was not significantly more common in first-time mothers, regardless of mode of delivery. However, CS was significantly more common in first-time mothers exposed to the pandemic. As seen in Table 3, exploratory subgroup analysis indicates this increase was confined to individuals who were induced or underwent pre-labour CS, suggesting that the decision threshold to deliver by CS following IOL or offering pre-labour CS may have altered during the pandemic. This is evidenced by the significant increases in Robson 2B births (pre-labour CS) from 1.85 to 2.39%, and the rate of CS in Robson 2A (births following induction of labour) from 35.55 to 38.61% during the pandemic-exposed period. Should the proportion of BMI *≥* 25 kg/m^2^ during pregnancy continue to rise, a compounding of the overall CS rate may be expected as success rates of a trial of labour after previous CS are lower for individuals with a high BMI [28]. 

Our exploratory subgroup analysis showed that macrosomia was associated with a significantly higher rate of CS in first-time mothers in both pre-pandemic and pandemic-exposed cohorts. Recognising and responding to risk factors for macrosomia other than maternal BMI *≥* 25 kg/m^2^ during pregnancy, such as gestational diabetes and excess gestational weight gain, will also likely play a role in reducing the primary CS rate. Future research on changing caesarean section rates should examine maternal birthing preferences, clinician decision making and health service factors driving this increase in CS among nulliparas in Australia.

A major strength of our study is the large multicentre dataset, capturing births from all 12 public maternity hospitals in Metropolitan Melbourne. We collected a complete birth cohort in a unique setting of strict COVID-19 lockdown restrictions but low maternal COVID-19 case-load. Our use of cLMP to define the pandemic-exposed cohort overcomes major methodological challenges of analysing time-dependent exposures. Another strength was the use of ITSA complimented by our single metric outcomes. By comparing forecasted trends in perinatal outcomes based on pre-pandemic trends, we could determine with greater confidence that the changes identified were associated with, rather than directly caused by, the pandemic.

Not all births within Metropolitan Melbourne were included in our analysis, as private hospital birth data were not available. There are major differences in maternal sociodemographic and obstetric factors between the public and private hospitals settings. In particular, CS rates are consistently higher in private hospitals compared with public hospitals [14]. We were not able to include gestational diabetes in our analysis due to coding inconsistencies in the source data. Furthermore, changes in the screening and treatment of gestational diabetes during the pandemic would have likely confounded this analysis.

## Conclusion

The pandemic period was associated with a greater proportion of maternal BMI ≥ 25 kg/m^2^ and CS in first-time mothers compared with the pre-pandemic period. These changes were continuations of pre-existing trends that were not accelerated by the pandemic. These trends are not likely to abate with the cessation of pandemic restrictions and have significant long-term implications for population health.

## Electronic supplementary material

Below is the link to the electronic supplementary material.

Supplementary Material 1

## Data Availability

Data is available upon reasonable request subject to HREC approval.

## Abbreviations

aRR: Adjusted relative risk
BMI: Body Mass Index
CI: Confidence intervals
cLMP: Calculated last menstrual period
COVID-19: Coronavirus Disease 2019
CS: Caesarean section
GA: Gestational age
IOL: Induction of labour
ISRAD: Index of Relative Socio-Economic Advantage and Disadvantage
ITSA: Interrupted time-series analysis
RANZCOG: Royal Australian and New Zealand College of Obstetricians and Gynecologists
MICE: Multiple imputation by changed equation
SD: Standard deviation
