# Supplementary material for: Trends in maternal body mass index, macrosomia and caesarean section in first-time mothers during the pandemic: a multicentre retrospective cohort study of 12 Melbourne public hospitals
Source: BMC Pregnancy Childbirth. 2024 Oct 28;24:706. doi: 10.1186/s12884-024-06908-y (PMC11514852; doi:10.1186/s12884-024-06908-y)
Supplement: Supplementary file 1 — Supplementary Material 1 [file 12884_2024_6908_MOESM1_ESM.docx]

**Supplementary File 1:** Detailed Methods

1. We conducted ITSA using the cLMP from October 30 2017 to 28 March 2022. Our intervention period started from cLMP of 4 November 2019. We employed ITSA using the 'itsa' suite of commands in Stata 18.[19] This approach used the Prais-Wintein generalized least-squares regression, accounting for autocorrelation of the residuals.[19]. We used sine and cosine functions to correct for seasonality.[19] The Prais-Winsten regression error terms followed a first-order autoregressive process.[19] We assessed the model's performance using Durbin-Watson statistics. Furthermore, we corrected our interrupted time-series model using the 'ssesearch' option in Stata 18, which minimized the sum-of-squares.[20] The reported coefficients included the pre-trend slope, exposure slope, and post-trend slope. Time-series figures were presented using coefficients in percentage (%) with 95% confidence intervals and p-values.

**Supplementary Table 1.** Characteristics of control and pandemic cohorts (All Robson groups)

| Characteristic | Pre-pandemic period | Pandemic period |
| --- | --- | --- |
| Total Births (%) | 66 906 (50.2) | 66 466 (49.8) |
| Macrosomic infants, n (%)*** | 6 207 (9.28) | 6 671 (10.04) |
| CS births, n (%)*** | 22 456 (33.56) | 23,523 (35.39) |
| Maternal Weight in Kg, mean (SD)*** | 70.3 (17.4) | 71.0 (17.5) |
| Maternal Height in cm, mean (SD) | 163.6 (7.0) | 163.7 (7.2) |
| Birth Weight in grams, mean (SD)** | 3337.4 (547.3) | 3347.6 (575.0) |
| Gestational Age at Birth weeks, mean (SD) | 39.02 (1.8) | 39.02 (1.8) |
| Maternal age group (years), n (%)*** |  |  |
| 18–24 | 8 508 (12.7) | 7 159 (10.8) |
| 25–29 | 15 203 (22.7) | 14 059 (21.2) |
| 30–34 | 26 083 (39.0) | 26 672 (40.2) |
| 35–39 | 14 114 (21.1) | 15 302 (23.1) |
| 40 or older | 2 998 (4.5) | 3 166 (4.8) |
| BMI Categories, n (%)* |  |  |
| <18 | 892 (1.4) | 782 (1.2) |
| 18–24 | 32 055 (48.5) | 29 963 (47.1) |
| 25–29 | 18 882 (28.2) | 18 846 (29.6) |
| 30–34 | 8 314 (12.6) | 8 232 (12.9) |
| 35–39 | 3 596 (5.4) | 3 557 (5.6) |
| >40 | 2 339 (3.5) | 2 268 (3.6) |
| Socio-economic status (IRSAD quintile), n (%)*** |  |  |
| 1 (most disadvantaged) | 14 683 (22.0) | 14 327 (21.6) |
| 2 | 9 886 (14.8) | 9 617 (14.5) |
| 3 | 15 982 (23.9) | 16 504 (24.8) |
| 4 | 14 749 (22.0) | 14 841 (22.3) |
| 5 (most advantaged) | 11 606 (17.4) | 11 177 (16.8) |
| Region of birth, n (%)*** |  |  |
| Australia and associated territories | 1 006 (1.5) | 1 061 (1.6) |
| Americas | 32 856 (49.3) | 34 085 (51.6) |
| North Africa and the Middle East | 3 420 (5.1) | 3 101 (4.7) |
| North-East Asia | 3 086 (4.6) | 2 314 (3.5) |
| North-West Europe | 1 993 (3.0) | 2 001 (3.0) |
| Oceania including New Zealand | 2 592 (3.9) | 2 402 (3.6) |
| South-East Asia | 5 850 (8.8) | 5 325 (8.1) |
| Southern and Central Asia | 12 411 (18.6) | 12 603 (19.1) |
| Southern and Eastern Europe | 1 439 (2.2) | 1 287 (2.0) |
| Sub-Saharan Africa | 2 040 (3.1) | 1 923 (2.9) |
| Smoking, n (%)** |  |  |
| Yes | 3 722 (5.6) | 3 134 (4.7) |
| Gestational age at birth, n (%)*** |  |  |
| 20 to 27 | 217 (0.3) | 255 (0.4) |
| 28 to 31 | 388 (0.6) | 422 (0.6) |
| 32 to 36 | 3 480 (5.2) | 3 281 (4.9) |
| 37 to 41 | 62 593 (93.6) | 62 150 (93.5) |
| 42+ | 228 (0.3) | 358 (0.5) |

IRSAD = Index of Relative Socio‐economic Advantage and Disadvantage

Statistical Significance * < 0.05, **<0.01, ***<0.001

N.B. Several categorical variable (%) may not sum to 100% due to rounding.

*All Robson groups*

There were a total of 66 906 births in the pre-pandemic cohort and 66 466 births in the pandemic-exposed cohort after exclusions and cohort selection criteria were applied (Figure 2). The maternal and neonatal characteristics of each cohort are shown in Supplemental Table 1. The proportion of all mothers with BMI ≥25kg/m2 was significantly higher in the pandemic cohort, although the absolute differences were small (51.7% vs 51.1%, p<0.001).

The rate of macrosomia in the overall obstetric population was higher among the pandemic-exposed cohort than in the pre-pandemic cohort (10.04% vs 9.23%, p<0.001) as was the overall CS rate (35.39% vs 33.56%, p<0.005). (Supplemental Table 1).
